# Could patents interfere with the development of a cardiovascular polypill?

**DOI:** 10.1186/s12967-016-0997-3

**Published:** 2016-08-18

**Authors:** Reed F. Beall, Jon-David R. Schwalm, Mark D. Huffman, Tara McCready, Salim Yusuf, Amir Attaran

**Affiliations:** 1 Population Health Program, University of Ottawa, One Stewart St, Ottawa, ON K1N 6N5 Canada; 2 Faculties of Law, University of Ottawa, One Stewart St, Ottawa, ON K1N 6N5 Canada; 3 Faculties of Medicine, University of Ottawa, One Stewart St, Ottawa, ON K1N 6N5 Canada; 4 Population Health Research Institute (PHRI), Hamilton Health Sciences, McMaster University, 237 Barton Street East, Hamilton, ON L8L 2X2 Canada; 5 Feinberg School of Medicine, Northwestern University, Arthur J. Rubloff Building, 420 East Superior Street, Chicago, IL 60611 USA

## Abstract

**Background:**

The Wellcome Trust, the World Health Organization, and cardiologists have advocated for the idea of a “polypill” containing multiple cardiovascular drugs to be co-formulated into a single pill for over a decade. Some cardiologists have asserted that the drugs commonly considered for inclusion into such a polypill are older and therefore free of patent protection. We tested this assertion. This project was requested by the World Heart Federation (WHF).

**Methods, data and materials:**

Two cardiologists from the WHF provided a list of 48 cardiovascular drugs for evaluation. We designated the United States and Canada as the base jurisdictions for this patent study. We linked patent data from these countries’ national medicine patent registers to patent information in over 96 other countries using Derwent and INPADOC via Thomson Innovation. We expanded our study beyond the aforementioned data linkage through a systematic search of the World Intellectual Property Organization’s PatentScope, which was based primarily upon the drugs’ active ingredient names.

**Results:**

In the United States and Canada, eight of the drugs were only available in the patent-protected, brand name formulation in one or both countries. Another 21 drugs had relevant patents, but generic equivalents were nevertheless available. Only 19 drugs (40 %) appeared entirely post-patent. Broadening the co-formulation searches globally, the overwhelming majority of drugs (40/48) were mentioned in patent applications for cardiovascular drug combinations.

**Conclusion:**

The assertion that most of these cardiovascular drugs are post-patent is accurate, but only in the sense that many of the original patents on these active ingredients have expired and that generic alternatives are usually available. The landscape of patents covering novel (co-) formulations is far more complex, however. Most research and development for cardiovascular combination medicines are likely to be undertaken by companies whose original patents on the active ingredient will soon expire or have recently expired. Cardiologists looking to accelerate polypill development may consider approaching such companies to partner.

**Electronic supplementary material:**

The online version of this article (doi:10.1186/s12967-016-0997-3) contains supplementary material, which is available to authorized users.

## Background

There is a major gap between the prevalence of hypertension, and recourse to effective treatment, particularly in developing countries, where 80 % of the disease burden lies [1, 2]. To address this, many have called for simplifying both the prescribing of and adherence to treatment by co-formulating (i.e., combining) several drugs into a single “polypill,” rather than 3–7 pills taken individually [3–6]. Triple and even quadruple co-formulations have been developed for conditions such as HIV/AIDS and tuberculosis, and are credited with improved treatment outcomes [7, 8]. A number of clinical trials [2, 9, 10] and meta-analyses [11, 12] of different polypill co-formulations suggest that the same strategy can be helpful for the treatment of hypertension and for the primary and secondary prevention of cardiovascular disease (CVD) [13, 14]. A polypill can also improve patient adherence, and it can reduce the risk of adverse drug interactions in patients taking multiple medications [15]. Given the potential to reduce cardiovascular events and the associated cost of care, public investment into the development of a polypill has been shown to be cost-effective [16]. Indeed, the World Health Organization has been calling for the development of a polypill for over a decade [17].

But while there is large appetite from the public health community for a polypill, no such thing is commonplace in today’s global pharmaceutical market. Why is this? Are there patent barriers to market entry? Experts on the treatment of CVD have stated that the drugs under consideration for inclusion in cardiovascular polypill prototypes are no longer covered by patents [3, 6, 18], but this presumption has not been rigorously tested. A very recent study was published that investigated the patent situation on five cardiovascular medicines in the United States and Europe, but did not extend beyond these drugs and geographic regions [19]. Several publications, both academic [4, 20] and otherwise [21, 22], have rightly called for a broad and global understanding of the polypill patent situation. This article is intended to address this need. It is written for a broad audience while bearing in mind that this project was undertaken at the request of the World Heart Federation (WHF).

## Methods, supporting materials, and data availability

We began by independently consulting two expert cardiologists (JDRS, MDH)—who both participated in a workshop on the polypill endorsed by the WHF—on what drugs are of particular interest for co-formulating. We used the union of their drug lists (n = 48 drugs) as the focal point for this patent study.

As patent grants vary by country, it is necessary to designate a base legal jurisdiction for patent studies as a starting point for analysis. Consistent with other published methodologies [23–29], we set the United States and Canada as our base jurisdictions because medicine patents are uniquely prevalent there. These countries have large pharmaceutical markets, grant a high number of patents annually, and have strong infrastructure for enforcing those patents, making them particularly attractive for pharmaceutical suppliers.

Both countries have publicly available medicine patent registers—the United States Food and Drug Administration’s Orange Book [30] and Health Canada’s Patent Register [31]—that allow users to search by active ingredient name. We therefore searched by each active ingredient name in each database and then recorded the patent information retrieved, if any. We also recorded whether an equivalent generic product was available on the market for each drug using the Orange Book [30] and Health Canada’s Drug Product Database [32]—that is to say, whether the product had already been “genericized” in the United States and Canada respectively. We considered an equivalent to be a generic product with the identical active ingredient(s), (co-)formulation, and strength as the brand name one in question (i.e., the originator’s patented version).

Next, we consulted two commercial-grade international patent search databases covering over 96 countries—INPADOC [33] and Derwent [34]—via Thomson Innovation [35]. These databases group patent filings into “patent families” (i.e., sets of related patents), which is either done automatically by their relationship to an original priority application (as is the case in INPADOC [36]) or is done manually by patent analysts (as is the case in Derwent). Using the union of the patent family groupings of INPADOC and Derwent adds to the robustness of studies such as these, both in terms of the type of patents covered and the countries covered by them [23]. We entered the American and Canadian patent data from those North American medicine patent registers into Thomson Innovation and retrieved the international patent families for each drug. Reasoning that patent protection for each application is unlikely to extend longer than the standard 20-year period, we removed all patents with application filing dates earlier than 1 January 1995.

Thereafter, we reviewed the title and abstract of each “Basic” patent identified by Derwent (i.e., a patent representing the typical one contained within each family). We scored the type of protections typically covered by the patents contained in each family according to their proposal of a new co-formulation (i.e., drug combinations), a new compound (i.e., the active ingredient), a new formulation (e.g., extended release tablet or capsule), a new method of treatment (i.e., using the drug to treat specific conditions), and/or a new manufacturing process.

Both a strength and limitation of the above method is that all patents included are related to currently marketed products. To provide an impression of potentially relevant patent literature that may have been excluded, we conducted supplemental searches in the World Intellectual Property Organization’s (WIPO) PatentScope database [37]. This database contains applications filed by those seeking protection in many or all of the 148 national signatories to the Patent Cooperation Treaty. We built search algorithms to capture patent applications on combinations of the drugs on our list of 48 medicines. Our search protocols are included Additional file 1: Appendix S1.

All of the above patent searches were performed in May and June 2015. Note that there is no objective, definitive point at which such searches have identified all relevant patents. An expert judgment has to be made when to stop. Our results therefore should be taken as a preliminary appraisal, reflecting our search strategy, and should not be regarded by anyone seeking to commercialize these drugs as a substitute for obtaining independent legal advice. Our raw datasets are available in this article’s supplementary materials (Additional file 2).

## Results

### The drugs’ patent/genericized status as single formulations in the United States and Canada

**Fig. 1:**
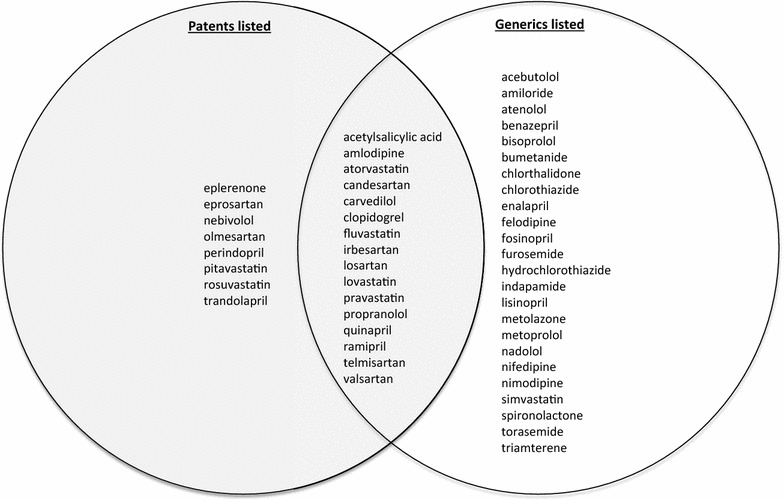
The 48 cardiovascular drugs *as single formulations* categorized by presence of generic competition and active patent listings in the United States or Canada

We found that eight of the 48 drugs (17 %) were available only as a brand name, patent-protected formulation in one of the base jurisdictions (the United States or Canada) (see Fig. 1). Olmesartan was the only drug available exclusively in the brand name in both countries.

Also shown in Fig. 1 are the 16 drugs (33 %) for which relevant patents were located in the United States or Canada, but had nevertheless been genericized. As for the remaining cardiovascular drugs (24 of 48 or 50 %), we located no valid patents and observed that the markets had indeed been genericized.

### The drugs’ patent/genericized status as co-formulations in the United States and Canada

**Fig. 2:**
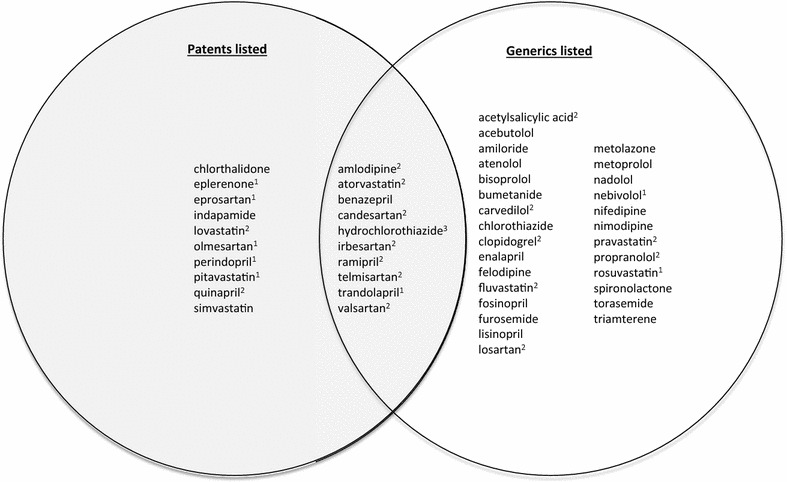
The 48 cardiovascular drugs in *FDCs* categorized by presence of generic competition and active patent listings in the United States or Canada. *1* This drug has one or more patents listed for it as a single formulation matched with an absence of generic alternatives in the concerned market (see Fig. 1). *2* While this drug has one or more patents listed for it as a single formulation, there are nevertheless generic alternatives available in that market (see Fig. 1)

As for fixed-dose combination (FDC) products containing one or more of the 48 drugs of interest, we found ten drugs (21 %) for which the only co-formulation(s) available in the United States or Canada was the patent-protected, brand name product (see Fig. 2). An additional ten drugs (21 %) were contained in one or more patented-protected FDCs, but had been genericized. For the majority of the cardiovascular drugs (28 of 48, or 58 %) investigated, however, we either located no patents using our methodology or observed that no co-formulations containing the drug in question were on the market.

### Patent filings by type of protection and by country

Table 1 shows the type of protections covered by the patent filings contained in the INPADOC and/or Derwent international patent families relating to the relevant US or Canadian marketed products in question. Patent protection on these drugs’ active ingredients was rare, but in some cases, not all patents had expired globally, even in North America.

**Table 1:** The 48 cardiovascular drugs categorized by type of patent protection and country

Drug	United States					Canada					Other countries				
	Patents^a^					Patents^a^					Patents^b^				
	Co-formulation	Compound	Formulation	Method	Process	Co-formulation	Compound	Formulation	Method	Process	Co-formulation	Compound	Formulation	Method	Process
Acebutolol															
Acetylsalicylic acid (aspirin)			X	X				X	X				16	16	
Amiloride															
Amlodipine	X		X	X	X	X					63	1	4	7	4
Atenolol															
Atorvastatin	X		X	X	X	X		X	X	X	50	4	53	39	39
Benazepril	X														
Bisoprolol															
Bumetanide															
Candesartan	X	X		X				X	X	X	1		4	10	9
Carvedilol			X	X	X			X	X	X			22	29	13
Chlorothiazide															
Chlorthalidone	X					X					31				
Clopidogrel			X		X			X		X		11	44		44
Enalapril												3			
Eplerenone	X		X	X		X		X	X		25	3	25	25	
Eprosartan	X		X	X	X	X		X	X	X	7	6	37	42	37
Felodipine															
Fluvastatin			X					X					36		
Fosinopril												2			
Furosemide															
Hydrochlorothiazide	X					X					60				
Indapamide	X					X					37				
Irbesartan	X		X			X		X			23	8	23		
Lisinopril												3			
Losartan			X		X							2			
Lovastatin	X		X					X			13	1	12	12	
Metolazone															
Metoprolol															
Nadolol															
Nebivolol			X					X				1	31	4	
Nifedipine															
Nimodipine															
Olmesartan	X	X	X	X	X	X		X			29	8	24	8	8
Perindopril			X	X	X	X		X	X	X	18		37	18	
Pitavastatin	X		X	X	X			X	X	X	13	2	17	17	10
Pravastatin				X					X			1		8	
Propranolol			X	X				X	X				10	10	
Quinapril			X	X			X					1	3	3	
Ramipril	X			X		X			X		34		34	35	
Rosuvastatin		X	X	X				X	X		6	50	33		
Simvastatin	X					X					19				
Spironolactone															
Telmisartan	X	X	X	X	X	X		X	X	X	49	6	35	17	36
Torasemide															
Trandolapril	X			X			X			X	3			2	
Triamterene															
Valsartan	X	X	X	X		X		X	X		42	7	36	36	
Total	18	5	20	18	10	14	2	18	14	9	523	120	536	338	200
	All countries														
Grand totals	555	127	574	370	219										

^a^A “X” means that at least one patent was found fitting into this category on the national patent register. The underline “X” denotes at least one patent was found in the INPADOC and/or Derwent patent family, but current legal status is unknown

^b^The numbers below indicate the number of jurisdictions (countries or regional agreements) covered by the patents in the INPADOC and Derwent patent families. Current legal status of these patents is unknown

By far, the most common type of protection afforded by these drugs’ respective patent families pertained to novel formulations or co-formulations. Patents applying to these categories were nearly five times more prevalent as compared to those on the active ingredient. Patents on using the drug as a method of treatment for cardiovascular disease or on a manufacturing process were also much more common than those on the active ingredient.

Nevertheless, as is shown in Table 1, we found no patents of any type whatsoever on 19 of these drugs in the United States and Canada, which cover all drug classes identified by the WHF cardiologists (i.e., statins, antiplatelets, angiotensin converting enzyme inhibitors, angiotensin II receptor blockers, calcium channel blockers, beta blockers, and diuretics).

### Searching for polypill co-formulation patents globally: WIPO PatentScope

Finally, to extend our analysis beyond the patents related to those listed in the American and Canadian medicine patent registers, we searched WIPO PatentScope for all patent applications that mention combinations of drugs within our list of 48 cardiovascular medicines. (see Additional file 1: Appendix S2 for four common approaches that we observed applicants had taken to construct their patent applications for cardiovascular FDCs.)

The overwhelming majority of the drugs (40 of 48, or 83 %) were identified in the co-formulation patent applications returned from WIPO PatentScope, either by active ingredient name or by drug class. Only eight drugs were unmentioned, all of which were older diuretics (amiloride, bumetanide, chlorthalidone, eplerenone, furosemide, metolazone, spironolactone, torasemide, triamterene). The remaining 3 (chlorothiazide, hydrochlorothiazide, indapamide) were identified as the diuretic of choice in many proposed co-formulations, especially hydrochlorothiazide.

## Discussion

Cardiologists’ perception [3, 6, 18] that the drugs being considered for CVD polypill co-formulations are post-patent has some empirical merit, but only in the sense that many of the original patents on the active ingredient(s) have expired and that the majority of these drugs have been genericized. This overlooks, however, that other forms of patent protection (i.e., formulation, co-formulation, method of treatment, manufacturing process) are more prevalent and can carry on for years after the expiration of the original patents on the active ingredient(s). We found that only 19 of the 48 drugs (40 %) were totally patent free in the base jurisdictions according to our methodology (see Table 1) and that most of the drugs (40 of 49, or 83 %) could be found on co-formulation patent applications filed through WIPO. When these secondary tiers of patenting are taken into account, it is more common to find patent filings than none whatsoever.

What is the significance of this finding for polypill advocates like the WHF who see promise in that treatment in developing countries? Below we discuss two perspectives on the patent system—for shorthand, the “competitive” versus “cooperative” perspectives—which differently inform two corresponding courses of action based on the findings and data presented in this report.

The “competitive” perspective is that patents represent strong, if temporary, barriers for others seeking to develop a technology and disseminate it widely. Patent owners possess exclusive rights to seek financial compensation in the law courts from those who infringe their technology. In this perspective, advocates of a CVD polypill should be prepared to deal with risk adverse pharmaceutical companies, who would likely not want to develop products that infringe upon these rights. Any patent is therefore a disincentive.

Based on this view of the patent system, our results, such as those in Table 1, may be read as a road map of existing obstacles to polypill co-formulating, while the non-shaded areas of Figs. 1 and 2 represent the patent-free freedom to operate. One could, then, work within the latter subset to propose a new cardiovascular FDC, which dodges the patent barriers. In doing so, advocates would be well advised to work with pharmaceutical firms with proven track records in the chemistry, manufacturing and controls aspects of making pharmaceuticals and with experience obtaining product registration. While all the major pharmaceutical companies have these capacities, some generics firms do as well. As of writing, one generics company (Ferrer) is already making FDCs that meet the requirements of stringent regulatory authorities in Europe, as are several India-based firms albeit without satisfying stringent regulatory authority standards [17, 38].

The “cooperative” perspective is that the patent system serves to incentivize new innovation, products and commercial activities. Patent owners acquire rights so as to make a business case for investment and commercialization. In this perspective, advocates of a CVD polypill should try to piggyback onto efforts that maximize the revenue pharmaceutical companies can obtain from their patent holdings, but in such a way that allowances are made for access to medicines in poorer countries.

Based on this view of the patent system, the shaded areas of Figs. 1 and 2 represent not barriers, but opportunities, because the patent holder’s monopoly brings with it a company that already has solved the technical and regulatory issues of their patented drug, and likely has the wherewithal and business interest to drive forward a new FDC including that drug. Indeed, evidence shows that companies become most receptive to develop new co-formulations as primary patents come close to expiring, so as to extend (or “evergreen”) market exclusivity [39]. See Table 2 for the age original patents on the active ingredients of the 48 drugs’ in descending order according to the Merck index [40]. There is empirical evidence that co-formulating is already happening for the most recently expired patents on the active ingredients: Daiichi Sankyo has recently introduced Tribenzor (amlodipine + hydrochlorothiazide + olmesartan), and Novartis has introduced Exforge HCT (the same, but substituting valsartan for olmesartan). Advocates would be well advised to create mutually beneficial arrangements with the pharmaceutical companies whose original patents on the active ingredient are drawing to an end, both to innovate polypills, and to bring these to market in developing countries at an affordable price. A clear lesson learned from the global campaigns for access to HIV/AIDS, malaria and other medicines is that companies can reconcile revenue maximization in rich countries with reduced revenue expectations or even philanthropic concessions in poor countries. They can do this by out-licensing their patents in the latter, refraining from enforcing their patents in certain regions, and/or offering substantial price reductions based on ability to pay (tiered pricing) [41–45]. Whatever access strategy is chosen, patents can be actively managed to serve as springboards for access campaigns, rather than managed as just barriers.

**Table 2:** Merck index active ingredient patent listing for the 48 cardiovascular drugs

INN	Latest patent grant year provided	International INPADOC family application date range	Patent numbers provided
Olmesartan	1997	1992–2011	EP503785, US5616599
Valsartan	1995	1991–2010	EP443983, US5399578
Candesartan	1993	1991–2006	EP459136, US5196444
Eprosartan	1993	1990–2001	EP403159, US5185351
Irbesartan	1993	1990–1999	WO9114679, US5270317
Atorvastatin	1993	1990–2007	EP409281, US5273995
Rosuvastatin	1993	1992–2003	EP521471, US5260440
Losartan	1992	1987–1999	EP253310, US5138069
Telmisartan	1992	1991–2011	EP502314
Fosinopril	1991	1988–1995	EP 304063, US5011930
Pitavastatin	1991	1988–1995	EP304063, US5011930
Trandolapril	1990	1981–1994	EP84164, US4933361
Clopidogrel	1989	1982–1998	EP99802, US4529596, EP281459, US4847265
Fluvastatin	1988	1983–1995	WO8402131, US4739073
Nebivolol	1987	1984–2004	EP145067, US4654362
Ramipril	1986	1982–1994	EP79022, US4587258
Amlodipine	1986	1983–1998	EP89167, US4572909
Perindopril	1985	1979–1993	EP49658, US4508729
Carvedilol	1985	1978–1994	DE2815926, US4503067
Eplerenone	1985	1984–2004	EP122232, US4559332
Simvastatin	1984	1980–1994	EP33538, US4444784
Benazepril	1983	1982–1993	EP72352, US4410520
Enalapril	1983	1979–1998	EP12401, US4374829
Lisinopril	1983	1979–1998	EP12401, US4374829
Quinapril	1982	1981–1996	EP49605, US4344949
Pravastatin	1982	1980–1996	DE3122499, US4346227
Felodipine	1981	1978–1994	EP7293, US4264611
Bisoprolol	1981	1976–1993	BE859425, US4258062
Lovastatin	1980	1978–1998	US4231938
Torasemide	1977	1974–1994	DE2516025, US4018929
Metoprolol	1976	1932–1977	DE2106209, US3998790
Nadolol	1976	1971–1979	DE2258995, US3935267, DE2421549
Nimodipine	1974	1971–1977	DE2117571, US3799934
Acebutolol	1974	1967–1974	ZA6808345, US3857952
Atenolol	1974	1969–1975	DE2007751, US3663607, US3836671
Bumetanide	1974	1968–1974	DE1964503, DE1964504, US3806534
Indapamide	1971	1968–1969	FR2003311, US3565911
Propranolol	1970	1962–1967	BE640312, BE640313, US3337628, US3520919
Nifedipine	1969	1967–1969	ZA6801482, US3485847
Amiloride	1967	1962–1981	BE639386, US3313813
Metolazone	1967	1966–1967	US3360518
Acetylsalicylic acid (aspirin)	1966	1959–1964	DE236196, US2890240, US3235583
Hydrochlorothiazide	1964	1962–1965	US3025292, US3163645, DE1163332, US3164588, US3043840
Triamterene	1963	1960–1964	US3081230
Chlorthalidone	1962	1957–1962	US3055904
Furosemide	1962	1959–1964	DE1122541, US305888
Spironolactone	1961	1960–1961	US3013012
Chlorothiazide	1957	1957	US2809194

We do not consider the “cooperative” and “competitive” scenarios mutually exclusive; rather they are complementary and should both be pursued. But both of them require that advocates make it extremely clear *exactly* which medicine combinations are best for an FDC. That choice has to be based on strong scientific consensus of the most clinically rational combinations, but not necessarily unanimity, and must strike a balance between the best therapeutic outcomes (for patient treatment success) and widespread suitability of the formulation (for population health coverage). Clear consensus is a true *sine qua non*, because whether seen through the eyes of a branded or generic company, advocates are calling on them to invest millions of dollars in FDC development and registration—and very simply put, companies will only sink that money when there is consensus guidance that says “the combination of A plus B plus C is satisfactory”, rather than equivocal guidance that says “the combination of A or B, plus C or D, plus E or F, but not if F is combined with C”.

The endorsement of advocates, or a coalition of advocates, to recommend a particular CVD co-formulation would likely appeal to drug makers and have a very significant impact on their willingness to invest. Since one would be endorsing a choice of co-formulation, and not a product, there is no conflict of interest in doing so. That would be a valuable step, whether pursuing a FDC built upon the “competitive” viewpoint of selecting only unpatented drugs over which nobody has exclusivity, or upon the “cooperative” viewpoint of selecting a drug precisely because it is patented and somebody has exclusivity. Our previous research in bringing low-cost new medicines to developing countries has shown that, depending upon circumstances, patent-centered strategies for improving access to medicines can be just as effective as patent-negating ones [41, 46, 47].

## Conclusion

Our study has tested the assertion that the drugs under consideration for polypill co-formulating are older, are post-patent, and have been genericized. For the original active ingredient patents, this is largely true, but our findings show that secondary patenting on these medicines is prevalent, and this includes large numbers co-formulation patents by generic and brand name companies alike.

We have suggested two strategies based on the empirical data provided by this study for global public health entities like the WHF who are in pursuit of developing a polypill, and these strategies can be undertaken simultaneously. Our impression, however, is that others attempting to advance polypill development have relied most heavily upon variants of the first strategy. We suggest a more balanced approach, set upon two parallel tracks, in which patents are viewed both as barriers and as opportunities, depending who the commercial partner is.

## Additional files

10.1186/s12967-016-0997-3 Co-formulation search criteria in WIPO PatentScope (run in May 2015). **Appendix S2.** Typology of search results from co-formulation searches in WIPO PatentScope.
10.1186/s12967-016-0997-3 Cardiovascular drug patent database.

## Abbreviations

CVD: Cardiovascular disease
FDC: Fixed-dose combination
INPADOC: *IN*ternational *PA*tent *DOC*umentation database, which is maintained by the European Patent Office
HIV/AIDS: Human immunodeficiency virus infection and acquired immune deficiency syndrome
WHF: The World Heart Federation
WIPO: The World Intellectual Property Organization
