# Supplementary material for: Could patents interfere with the development of a cardiovascular polypill?
Source: J Transl Med. 2016 Aug 18;14:242. doi: 10.1186/s12967-016-0997-3 (PMC4991009; doi:10.1186/s12967-016-0997-3)
Supplement: Supplementary file 1 — 10.1186/s12967-016-0997-3 Co-formulation search criteria in WIPO PatentScope (run in May 2015). Appendix S2. Typology of search results from co-formulation searches in WIPO PatentScope. [file 12967_2016_997_MOESM1_ESM.docx]

Appendix 1. Co-formulation search criteria in WIPO PatentScope (run in May 2015)

1. Search strategy 1 for co-formulation applications in PatentScope

PD:[15.04.2005 TO 15.04.2015] AND FP: ("co-" OR combi* OR coform* OR fixed) AND FP:("acebutolol" OR "acetylsalicylic" OR "amiloride" OR "amlodipine" OR "aspirin" OR "atenolol" OR "atorvastatin" OR "atorvastatin" OR "benazepril" OR "bisoprolol" OR "bumetanide" OR "candesartan" OR "carvedilol" OR "chlorothiazide" OR "chlorthalidone" OR "clopidogrel" OR "enalapril" OR "eplerenone" OR "eprosartan" OR "felodipine" OR "fluvastatin" OR "fosinopril" OR "furosemide" OR "hydrochlorothiazide" OR "indapamide" OR "irbesartan" OR "lisinopril" OR "losartan" OR "lovastatin" OR "metolazone" OR "metoprolol" OR "nadolol" OR "nebivolol" OR "nifedipine" OR "nimodipine" OR "olmesartan" OR "perindopril" OR "pitavastatin" OR "pravastatin" OR "propranolol" OR "quinapril" OR "ramipril" OR "rosuvastatin" OR "rosuvastatin" OR "simvastatin" OR "spironolactone" OR "telmisartan" OR "torasemide" OR "trandolapril" OR "triamterene" OR "valsartan") AND EN_AB: ("co-" OR combi* OR coform* OR fixed) AND EN_AB:("acebutolol" OR "acetylsalicylic" OR "amiloride" OR "amlodipine" OR "aspirin" OR "atenolol" OR "atorvastatin" OR "atorvastatin" OR "benazepril" OR "bisoprolol" OR "bumetanide" OR "candesartan" OR "carvedilol" OR "chlorothiazide" OR "chlorthalidone" OR "clopidogrel" OR "enalapril" OR "eplerenone" OR "eprosartan" OR "felodipine" OR "fluvastatin" OR "fosinopril" OR "furosemide" OR "hydrochlorothiazide" OR "indapamide" OR "irbesartan" OR "lisinopril" OR "losartan" OR "lovastatin" OR "metolazone" OR "metoprolol" OR "nadolol" OR "nebivolol" OR "nifedipine" OR "nimodipine" OR "olmesartan" OR "perindopril" OR "pitavastatin" OR "pravastatin" OR "propranolol" OR "quinapril" OR "ramipril" OR "rosuvastatin" OR "rosuvastatin" OR "simvastatin" OR "spironolactone" OR "telmisartan" OR "torasemide" OR "trandolapril" OR "triamterene" OR "valsartan") AND EN_CL: ("co-" OR combi* OR coform* OR fixed) AND EN_CL:("acebutolol" OR "acetylsalicylic" OR "amiloride" OR "amlodipine" OR "aspirin" OR "atenolol" OR "atorvastatin" OR "atorvastatin" OR "benazepril" OR "bisoprolol" OR "bumetanide" OR "candesartan" OR "carvedilol" OR "chlorothiazide" OR "chlorthalidone" OR "clopidogrel" OR "enalapril" OR "eplerenone" OR "eprosartan" OR "felodipine" OR "fluvastatin" OR "fosinopril" OR "furosemide" OR "hydrochlorothiazide" OR "indapamide" OR "irbesartan" OR "lisinopril" OR "losartan" OR "lovastatin" OR "metolazone" OR "metoprolol" OR "nadolol" OR "nebivolol" OR "nifedipine" OR "nimodipine" OR "olmesartan" OR "perindopril" OR "pitavastatin" OR "pravastatin" OR "propranolol" OR "quinapril" OR "ramipril" OR "rosuvastatin" OR "rosuvastatin" OR "simvastatin" OR "spironolactone" OR "telmisartan" OR "torasemide" OR "trandolapril" OR "triamterene" OR "valsartan")

2. Search strategy 2 for co-formulation applications in PatentScope with specifications for CVD

PD:[15.04.2005 TO 15.04.2015] AND FP:("co-" OR combi* OR synerg* OR symb* OR mix* OR form* OR composition* OR fixed OR oral* OR dos* OR single OR daily OR day OR once*) AND EN_AB:("co-" OR combi* OR synerg* OR symb* OR mix* OR form* OR composition* OR fixed OR oral* OR dos* OR single OR daily OR day OR once*) AND EN_CL:("co-" OR combi* OR synerg* OR symb* OR mix* OR form* OR composition* OR fixed OR oral* OR dos* OR single OR daily OR day OR once*) AND FP:(CVD OR cardiovascular OR hypertens* OR heart OR pulmonary OR angina OR arter* OR coronary OR angiotensin OR atherosclerosis) AND EN_AB:(CVD OR cardiovascular OR hypertens* OR heart OR pulmonary OR angina OR arter* OR coronary OR angiotensin OR atherosclerosis) AND EN_CL:(CVD OR cardiovascular OR hypertens* OR heart OR pulmonary OR angina OR arter* OR coronary OR angiotensin OR atherosclerosis) AND FP:("acebutolol" OR "acetylsalicylic" OR "amiloride" OR "amlodipine" OR "aspirin" OR "atenolol" OR "atorvastatin" OR "atorvastatin" OR "benazepril" OR "bisoprolol" OR "bumetanide" OR "candesartan" OR "carvedilol" OR "chlorothiazide" OR "chlorthalidone" OR "clopidogrel" OR "enalapril" OR "eplerenone" OR "eprosartan" OR "felodipine" OR "fluvastatin" OR "fosinopril" OR "furosemide" OR "hydrochlorothiazide" OR "indapamide" OR "irbesartan" OR "lisinopril" OR "losartan" OR "lovastatin" OR "metolazone" OR "metoprolol" OR "nadolol" OR "nebivolol" OR "nifedipine" OR "nimodipine" OR "olmesartan" OR "perindopril" OR "pitavastatin" OR "pravastatin" OR "propranolol" OR "quinapril" OR "ramipril" OR "rosuvastatin" OR "rosuvastatin" OR "simvastatin" OR "spironolactone" OR "telmisartan" OR "torasemide" OR "trandolapril" OR "triamterene" OR "valsartan") AND EN_AB:("acebutolol" OR "acetylsalicylic" OR "amiloride" OR "amlodipine" OR "aspirin" OR "atenolol" OR "atorvastatin" OR "atorvastatin" OR "benazepril" OR "bisoprolol" OR "bumetanide" OR "candesartan" OR "carvedilol" OR "chlorothiazide" OR "chlorthalidone" OR "clopidogrel" OR "enalapril" OR "eplerenone" OR "eprosartan" OR "felodipine" OR "fluvastatin" OR "fosinopril" OR "furosemide" OR "hydrochlorothiazide" OR "indapamide" OR "irbesartan" OR "lisinopril" OR "losartan" OR "lovastatin" OR "metolazone" OR "metoprolol" OR "nadolol" OR "nebivolol" OR "nifedipine" OR "nimodipine" OR "olmesartan" OR "perindopril" OR "pitavastatin" OR "pravastatin" OR "propranolol" OR "quinapril" OR "ramipril" OR "rosuvastatin" OR "rosuvastatin" OR "simvastatin" OR "spironolactone" OR "telmisartan" OR "torasemide" OR "trandolapril" OR "triamterene" OR "valsartan") AND EN_CL:("acebutolol" OR "acetylsalicylic" OR "amiloride" OR "amlodipine" OR "aspirin" OR "atenolol" OR "atorvastatin" OR "atorvastatin" OR "benazepril" OR "bisoprolol" OR "bumetanide" OR "candesartan" OR "carvedilol" OR "chlorothiazide" OR "chlorthalidone" OR "clopidogrel" OR "enalapril" OR "eplerenone" OR "eprosartan" OR "felodipine" OR "fluvastatin" OR "fosinopril" OR "furosemide" OR "hydrochlorothiazide" OR "indapamide" OR "irbesartan" OR "lisinopril" OR "losartan" OR "lovastatin" OR "metolazone" OR "metoprolol" OR "nadolol" OR "nebivolol" OR "nifedipine" OR "nimodipine" OR "olmesartan" OR "perindopril" OR "pitavastatin" OR "pravastatin" OR "propranolol" OR "quinapril" OR "ramipril" OR "rosuvastatin" OR "rosuvastatin" OR "simvastatin" OR "spironolactone" OR "telmisartan" OR "torasemide" OR "trandolapril" OR "triamterene" OR "valsartan")

3. Search strategy for polypill prototype applications in PatentScope with specifications

PD:[15.04.1995 TO 15.04.2015] AND FP: ("co-" OR combi* OR coform* OR fixed) AND FP:(CVD OR cardiovascular* OR hypertens* OR heart* OR pulmonary OR angina OR arter* OR coronary OR angiotensin* OR atherosclerosis) AND EN_CL:(anti-platelet OR aspirin* OR "ASA" OR acetylsalicylic* OR clopidogrel*) AND EN_CL:(*statin* OR atorvastatin OR simvastatin OR simvastatin OR rosuvastatin OR pravastatin OR lovastatin OR fluvastatin OR pitavastatin) AND EN_CL:(“ACE” OR angiotensin* OR *pril OR benazepril OR fosinopril OR enalapril OR lisinopril OR ramipril OR perindopril OR trandolapril OR quinapril) AND EN_CL:(“ARB” OR angiotensin* OR “AT1” OR *sartan OR losartan OR irbesartan OR valsartan OR candesartan OR telmisartan OR olmesartan) AND EN_CL:(“CCB” OR *calcium* channel* OR blocker* OR *dipine OR amlodipine OR felodipine OR nimodipine OR nifedipine) AND EN_CL:(beta OR blocker OR “BB” OR *olol OR atenolol OR bisoprolol OR nadolol OR nebivolol OR acebutolol OR metoprolol OR propranolol OR carvedilol) AND EN_CL:(diuretic* OR hydrochlorothiazide OR indapamide OR chlorthalidone OR furosemide OR bumetanide OR torasemide OR amiloride OR triamterene OR chlorothiazide OR spironolactone OR eplerenone OR metolazone)

Appendix 2. Typology of search results from co-formulation searches in WIPO PatentScope

Our iterative process used three searches (see Appendix 1), incorporating increasingly specific criteria to locate patent applications that describe cardiovascular co-formulations made from combinations of the 48 drugs of interest; these searches returned 211, 178, 14 publications respectively. We observed that the patent applications were constructed according to one of the following four approaches below. Patents have been granted for applications falling into all four of these categories (especially those naming very specific fixed dose combinations), but not consistently so. The final category, which is the most vague and far-reaching, appeared to be the most difficult to patent; while we observed grants on such applications in some jurisdictions, we saw several denials in other places.

| Application approach | Example | Analysis |
| --- | --- | --- |
| One specific active ingredient with *any* other active ingredient | WO/2010/036600 by Merck states that its formulation patent application applies to “atorvastatin as the only active agent or combined with one or more additional active agents.” | Should these patents be granted in the relevant country, these patents may block development. It is often the case, however, that the original compound and/or formulation patent has already (or should have already) laid out this claim and has since expired. |
| Two or more specific active ingredient combinations | WO/2013/100630 by Hanmi Pharmaceuticals proposes a FDC of amlodipine + hydrochlorothiazide + valsartan. An extreme example is Cipla’s WO/2009/087410, which lists all 33 ingredients under consideration for co-formulations (8 of these ingredients were on our list) (note that this patent application has since been abandoned). | These can be handled as traditional FDC patents. Should such a patent be granted in the relevant jurisdiction and should it include the same ingredients in one’s polypill recipe or be reasonably similar, it is worth following up and consulting legal advice before proceeding. Many FDC patents are proposed and later abandoned due to factors like manufacturing feasibility. |
| One specific active ingredient with an ingredient from one or more other ingredient classification(s) | Wockhardt Limited’s WO/2013/084089 states: “…fixed dose combination of metoprolol in extended release form and one or more calcium channel blocker, angiotensin II receptor blocker or angiotensin converting enzyme inhibitor along with one or more rate controlling excipient.” | Other classifications may be rather specific (e.g., a HMG-CoA reductase inhibitor (statins)) or more general (e.g., at least one other active agent, wherein the at least one other active agent is a cardiovascular agent). The more specific are more concerning. Should such a patent be granted in the relevant jurisdiction with a fairly specific and similar prototype description, further legal investigation is necessary. |
| One or more active ingredient classifications with other classifications for treating specific conditions | Novartis’ WO/2006/116435 is illustrative, describing the invention as including “…at least one therapeutic agent selected from the group consisting of: (1) an ACE inhibitor, or a pharmaceutically acceptable salt thereof; (2) an angiotensin II receptor blocker, or a pharmaceutically acceptable salt thereof; (3) a diuretic, or a pharmaceutically acceptable salt thereof; (4) a calcium channel blocker (CCB), or a pharmaceutically acceptable salt thereof; (5) a beta-blocker, or a pharmaceutically acceptable salt thereof; (6) a platelet aggregation inhibitor, or a pharmaceutically acceptable salt thereof; (7) a cholesterol absorption modulator, or a pharmaceutically acceptable salt thereof; (8) a HMG-Co-A reductase inhibitor, or a pharmaceutically acceptable salt thereof; and (9) a high density lipoprotein (HDL) increasing compound, or a pharmaceutically acceptable salt thereof.” | Specific categories are named with several active ingredients as examples (e.g., a statin such as atorvastatin, lovastatin, or simvastatin). They also tend to describe the combination as treatment for one or more target illnesses. These applications are clearly filed by those in pursuit of developing a polypill. Should these applications closely describe the one’s polypill prototype for the same medical condition and be granted in the relevant jurisdiction, it would be important to follow up and seek legal advice. Simple adjustments to already patented combinations may be considered too obvious for a patent to be granted. |
